# Phylogenetic assessment reveals continuous evolution and circulation of pigeon-derived virulent avian avulaviruses 1 in Eastern Europe, Asia, and Africa

**DOI:** 10.1186/s12917-017-1211-4

**Published:** 2017-09-26

**Authors:** Mahmoud Sabra, Kiril M. Dimitrov, Iryna V. Goraichuk, Abdul Wajid, Poonam Sharma, Dawn Williams-Coplin, Asma Basharat, Shafqat F. Rehmani, Denys V. Muzyka, Patti J. Miller, Claudio L. Afonso

**Affiliations:** 1 Department of Poultry Diseases, Faculty of Veterinary Medicine, South Valley University, Qena, 83523 Egypt; 2 Exotic and Emerging Avian Viral Diseases Research Unit, Southeast Poultry Research Laboratory, US National Poultry Research Center, Agricultural Research Service, USDA, 934 College Station Road, Athens, GA 30605 USA; 3 National Scientific Center Institute of Experimental and Clinical Veterinary Medicine, 83 Pushkinskaya Street, Kharkiv, 61023 Ukraine; 4 Quality Operations Laboratory (QOL), University of Veterinary and Animal Sciences, Syed Abdul Qadir Jilani Road, Lahore, 54000 Pakistan; 5 Institute of Biochemistry and Biotechnology, University of Veterinary and Animal Sciences, Syed Abdul Qadir Jilani Road, Lahore, 54000 Pakistan

**Keywords:** Newcastle disease virus, NDV, Pigeons, Genotype VI, rRT-PCR, Mismatches, Evolution, Next-generation sequencing

## Abstract

**Background:**

The remarkable diversity and mobility of Newcastle disease viruses (NDV) includes virulent viruses of genotype VI. These viruses are often referred to as pigeon paramyxoviruses 1 because they are normally isolated and cause clinical disease in birds from the *Columbidae* family. Genotype VI viruses occasionally infect, and may also cause clinical disease in poultry. Thus, the evolution, current spread and detection of these viruses are relevant to avian health.

**Results:**

Here, we describe the isolation and genomic characterization of six Egyptian (2015), four Pakistani (2015), and two Ukrainian (2007, 2013) recent pigeon-derived NDV isolates of sub-genotype VIg. These viruses are closely related to isolates from Kazakhstan, Nigeria and Russia. In addition, eight genetically related NDV isolates from Pakistan (2014–2016) that define a new sub-genotype (VIm) are described. All of these viruses, and the ancestral Bulgarian (*n* = 2) and South Korean (*n* = 2) viruses described here, have predicted virulent cleavage sites of the fusion protein, and those selected for further characterization have intracerebral pathogenicity index assay values characteristic of NDV of genotype VI (1.31 to 1.48). A validated matrix gene real-time RT-PCR (rRT-PCR) NDV test detect all tested isolates. However, the validated rRT-PCR test that is normally used to identify the virulent fusion gene fails to detect the Egyptian and Ukrainian viruses due to mismatches in primers and probe. A new rapid rRT-PCR test to determine the presence of virulent cleavage sites for viruses from sub-genotypes VIg was developed and evaluated on these and other viruses.

**Conclusions:**

We describe the almost simultaneous circulation and continuous evolution of genotype VI Newcastle disease viruses in distant locations, suggesting epidemiological connections among three continents. As pigeons are not migratory, this study suggests the need to understand the possible role of human activity in the dispersal of these viruses. Complete genomic characterization identified previously unrecognized genetic diversity that contributes to diagnostic failure and will facilitate future evolutionary studies. These results highlight the importance of conducting active surveillance on pigeons worldwide and the need to update existent rapid diagnostic protocols to detect emerging viral variants and help manage the disease in affected regions.

**Electronic supplementary material:**

The online version of this article (10.1186/s12917-017-1211-4) contains supplementary material, which is available to authorized users.

## Background

Virulent Newcastle disease viruses (NDV), synonymous with avian avulaviruses 1 (AAvV-1), are the causative agents of Newcastle disease (ND). Newcastle disease is one of the most important infectious diseases of poultry because of its worldwide distribution and devastating economic effects for the poultry industry [1–3]. The disease is highly contagious, presents high morbidity and mortality, and is classified as a notifiable disease by the World Organisation for Animal Health (OIE) [3]. AAvV-1 (along with another 12 serotypes, namely AAvV 2–13) belongs to genus *Avulavirus* of the family *Paramyxoviridae* [4, 5]. Several additional AAvV, have been recently proposed as potential new serotypes [6–9]. Avian avulaviruses 1 are enveloped, have a single stranded, non-segmented, negative sense RNA genome with helical capsid symmetry [10]. Three different genomic sizes (15,186 nucleotides [nt], 15,192 nt and 15,198 nt) have been identified so far, and all known AAvV-1 are divided into two major genetic groups, class I and class II [11]. Currently, AAvV-1 isolates are classified into 18 class II and one class I genotypes based on the complete coding sequences of the fusion protein [12, 13]. For the purpose of this work, the taxa name Newcastle disease virus will be used.

Pigeons may be infected with NDV of all genotypes, but are particularly susceptible to the genotype VI genetic variants, also referred to as pigeon paramyxovirus 1 (PPMV-1). These genotype VI viruses are mainly isolated from Rock Pigeons (*Columba livia*), but also have been isolated from other members of the family *Columbidae,* e.g. feral Eurasian Collared Doves (*Streptopelia decaocto*) [14]. Viruses of genotype VI are endemic in the pigeon population throughout the world, and can be distinguished as being variants of NDV by the patterns produced in a hemagglutination inhibition (HI) assay [15, 16] when tested with a panel of monoclonal antibodies. Genotype VI viruses have evolved rapidly since their emergence in the Middle East during the 1960s and are currently divided into at least eleven sub-genotypes (and one more putative for a group of viruses identified in Ethiopia), namely VIa-VIj (respectively VIl) [13, 17].

Commonly, these viruses are an example of viruses that have a cleavage site motif that is generally associated with virulent viruses. However, most of them are considered to be of intermediate or low virulence for chickens, as assessed through the intracerebral pathogenicity index (ICPI) test [18, 19]. Nevertheless, their pathogenicity might be increased after passage in poultry species [20, 21].

Diagnostic testing and rapid detection of NDV are important steps in controlling an ND outbreak. Virus isolation in specific pathogen free (SPF) embryonated chicken eggs (ECE), followed by identification using hemagglutination (HA) and HI assays with a NDV-monospecific antiserum [3] is considered “gold standard” for NDV diagnostics. This approach is time-consuming and laborious and often requires up to ten days [22]. Molecular diagnostics assays are a viable alternative to classical diagnostic assays and are widely used. Several protocols for the detection of NDV by reverse-transcription PCR (RT-PCR) have been published in the last decade [23]. Real-time RT-PCR (rRT-PCR) is a rapid screening assay allowing for detection and pathotyping of NDV directly from diagnostic specimens and different protocols based on the use of hydrolysis probes, SybrGreen or LUX primers, have been published [24–26]. Real-time RT-PCR is highly dependent on genetic similarity between the primers/probe and the target genome, and protocols have to be updated as the genomes of pathogens accumulate mutations over time.

To improve our understanding of the distribution and evolution of NDV of genotype VI, viruses of this genotype circulating in five countries from 1982 to 2016 were isolated and studied. For this purpose, the following studies were done: i) isolation and biological and phylogenetic characterization of genotype VI viruses from different geographical locations; ii) analyses of the complete fusion protein gene coding sequences and complete genome sequences obtained from the studied viruses; iii) evaluation of their epidemiological relation to other circulating NDVs; and iv) optimization of rRT-PCR diagnostic test for detection of virulent variants of sub-genotype VIg as a result of identified failure of the current validated diagnostic protocol.

## Methods

### Sample collection and isolates background data

The samples and viruses studied here were collected from different species (pigeons, chickens and quail) in Egypt, Pakistan, South Korea, Ukraine and Bulgaria, representing three different continents (Africa, Asia and Europe). During April 2015, one hundred sixty-seven (*n* = 167) oropharyngeal and cloacal/fecal swabs were collected in Egypt from apparently healthy Rock Pigeons. Samples were collected from pigeon lofts in El Fayoum and Qena provinces, and a live bird market (LBM) in Cairo named Souq al-Goma’a (also named Souq Sayeda Aisha or Friday Market). The swabs were placed immediately into tubes with 3 ml of brain-heart-infusion broth (Difco, New Zealand) supplemented with penicillin G (10,000 IU /ml), amphotericin B (20 μg/ml), and gentamycin (1000 μg/ml). After collection, the samples were labelled, stored on ice, transported to the lab where kept frozen at −76 °C, and shipped on dry ice to the Southeast Poultry Research Laboratory (SEPRL) of the United States Department of Agriculture (USDA). Additionally, three more samples were collected from pigeons in Pakistan in 2015 and shipped to SEPRL, USDA. Six additional NDV from repositories in Bulgaria (*n* = 2), Ukraine (*n* = 2), and South Korea (*n* = 2), were also sent to SEPRL for further characterization. The Bulgarian and the Ukrainian viruses were passaged in eggs 3 times and 2 times, respectively. The Korean isolates were passaged 2 times at SEPRL and passage information was missing prior to their receiving. Nine more NDV, isolated between 2014 and 2016 from healthy and diseased pigeons (pet and zoo birds), were studied at the Quality Operations Laboratory (QOL) at the University of Veterinary and Animal Sciences (UVAS), Lahore, Pakistan.

### Virus isolation, virus propagation and intracerebral pathogenicity index test

Initial screening of all Egyptian samples employing the NDV and avian influenza matrix gene rRT-PCR assays [24, 27], revealed that out of 167 oropharyngeal and cloacal swabs, 71 samples had cycle threshold (Ct) values ≤35 (40.3%) in the NDV assay and all samples were negative in the avian influenza assay. At SEPRL, thirty-one (selected to achieve representativeness of all locations) of the 71 rRT-PCR positive Egyptian samples and three Pakistani samples from 2015 (designated 21A, 22A and 25A) were selected for further studies and inoculated into 9-to-11-day-old SPF ECE, following standard procedures [28]. The SPF ECE and chickens used to characterize these viruses were from the SEPRL SPF White Leghorn flock. The allantoic fluids from both eggs with embryo mortality and embryos alive at the end of the incubation period were collected and tested by hemagglutination assay. All the hemagglutinating agents were confirmed as NDV using a HI assay with NDV specific antiserum [3]. Additionally, The NDV obtained from repositories in Ukraine, Bulgaria and South Korea were propagated into 9-to-11-day-old SPF ECE following the same procedures [28]. Assessment of the virulence in vivo of three selected viruses was done by the ICPI test using one-day-old SPF chickens following established procedures [3].

### Complete fusion protein gene sequencing

RNA from the two South Korean isolates was extracted from infected allantoic fluid using TRIzol LS Reagent (Invitrogen, Carlsbad, CA, USA) following the manufacturer’s instructions at SEPRL. RT-PCR was performed to amplify the complete F gene using the Superscript™ III One-step RT-PCR kit with Platinum Taq DNA polymerase (Invitrogen, Carlsbad, CA, USA), per manufacturer’s instructions using a set of F-gene specific primers (4331F/5090R, MSF1/NDVR2, 4927F/5673R and 5491F/6341R) [29] (see Additional file 1: Table S1). The PCR amplicons were processed and sequenced as described previously [30]. RNA isolation and nucleotide sequencing of nine Pakistani viruses were performed in Quality operations laboratory at University of Veterinary and Animal Science in Pakistan as follows: RNA extraction and RT-PCR F-gene amplification were performed as described above using a set of F-gene specific primers (see Additional file 1: Table S1). The amplicons were electrophoresed using 1% agarose gel and purified using QIAquick® Gel Extraction Kit (Qiagen, Valencia, CA, USA). The purified products were sequenced using the ABI 3130 automated sequencer (Applied Biosystem Inc., Foster City, CA, USA), as described previously [30].

### Complete genome sequencing using next-generation sequencing (NGS)

Total viral RNA of six Egyptian (positive in virus isolation assay), two Ukrainian, three Pakistani, and two Bulgarian viruses were extracted from the infected allantoic fluids using QIAmp® Viral RNA Mini Kit (Qiagen, USA) according to manufacturer’s instructions. The recovered RNA was quantified using the Qubit® RNA HS Assay Kit (Life Technologies, USA) in the Qubit® Fluorometer instrument (Invitrogen, USA). Newcastle disease virus RNA was captured and enriched using Sera-Mag beads (GE Healthcare Life Sciences, USA), and three biotin-labeled oligonucleotide probes targeting three different positions in the NDV genome: 1) 8-AGA GAA TCT GTG AGG TAC GA/3Bio/, 2) 5905-TTC TCA AGT CAT CGT GAC AG/3Bio/, and 3) 12226-CCC TGC ATC TCT CTA CAG/3Bio/. Reverse transcription was performed using the Moloney Murine Leukemia Virus Reverse Transcriptase (M-MLV RT) kit (Thermo Scientific, USA) according to the manufacturer’s instructions. The cDNA products were recovered and purified using the Agencourt® RNAClean® XP beads (Beckman Coulter, USA) according to the manufacturer’s instructions and quantified using Qubit® ssDNA Assay Kit (Thermo Fisher Scientific, USA) in the Qubit instrument. The purified cDNA products were tagmented and amplified for NGS by using 1 ng/5 μl (0.25 ng/μl in water) of the cDNA product employing the Nextera XT DNA Library Preparation Kit (Illumina, USA) following manufacturer’s protocol. The two Bulgarian samples were processed using the KAPA Stranded RNA-Seq Library Preparation Kit for Illumina platforms (Kapa Biosystems, USA) according to the manufacturer’s instructions.

The distribution size and concentration of DNA in the prepared libraries were checked on a Bioanalyzer 2100 and Qubit instrument using Agilent High Sensitivity DNA Kit (Agilent Technologies, Germany) and Qubit® dsDNA HS Assay Kit (Life Technologies, USA), respectively. Paired-end sequencing (2 × 250 base pairs) of the generated libraries was performed on an Illumina MiSeq instrument using the 500 cycle MiSeq Reagent Kit version 2 (Illumina, USA). Raw sequence data were analyzed and assembled using MIRA version 3.4.1 [31] within a customized workflow on the Galaxy platform [32] as described previously [33]. Short internal gaps at the 3′ UTR of the nucleoprotein gene were closed using Sanger technology with primers designed using the sequences obtained from NGS (see Additional file 1: Table S1). The 5′ and 3′ ends of the genomes reported here were sequenced and confirmed as described previously [34].

### Collection of sequences and phylogenetic analyses

All available NDV complete genome and complete fusion protein gene coding sequences were downloaded from GenBank and curated, resulting in two large datasets (*n* = 331 and *n* = 1406, respectively). Together with the sequences obtained in the current study, all sequences were aligned using Multiple Alignment with Fast Fourier Transformation (MAFFT v7.017) [35] as implemented in the Geneious software v8.1.4 [36]. The datasets were used for two preliminary maximum likelihood phylogenetic analyses using MEGA6 [37]. Based on evolutionary relatedness to the viruses sequenced here, two smaller datasets (including newly obtained sequences) of complete fusion protein gene coding sequences (*n* = 82) (see Additional file 1: Table S2) and complete genome sequences (*n* = 83) (see Additional file 1: Table S3) were parsed from the larger datasets. Sequences of selected representative isolates from other NDV genotypes were also included in each of the datasets. The coding regions of the complete genome and complete fusion gene were used to construct final phylogenetic trees using MEGA6. To select best-fit substitution model, the Bayesian Information Criterion (BIC) and corrected Akaike Information Criterion (AICc) values were estimated using MEGA6. The General Time Reversible (GTR) model as implemented in MEGA6 with a discrete gamma distribution (4 categories [+G, parameter = 0.6519 for the full fusion gene tree and 0.5984 for the complete genome tree]) with 1000 bootstrap replicates was used in all data analysis. The phylogenetic trees were visualized and edited using tree explorer implemented in MEGA6 and branch lengths are proportional to the differences between the isolates. The evolutionary distances were inferred using MEGA6 and showed as the average number of base substitutions per site. Analyses were conducted using the maximum composite likelihood model [38] with a gamma distribution (shape parameter = 1) of rate variation among sites. For all analyses, the codon positions included were the 1st, 2nd, 3rd, and noncoding and positions containing gaps and missing data were eliminated from the datasets. The classification criteria proposed by Diel et al. for naming sub-genotypes and genotypes were followed in the current study [12].

### Real-time reverse transcription polymerase chain reaction (rRT-PCR)

Allantoic fluids from all of the samples studied at SEPRL were subjected to the USDA-validated matrix gene rRT-PCR (M-gene assay), as well as to the USDA-validated fusion gene rRT-PCR assay (F-gene assay) described previously by Wise et al. [24]. An additional test was performed using pigeon-specific fusion protein gene assay as described previously by Kim et al. [39]. AgPath one-Step rRT-PCR Reagents (Thermo Scientific, USA) was used and the reactions were carried out in the Cepheid Real-Time Thermal Cycler (Life Science, USA). The samples that had a Ct value ≤35 were considered positive in all assays. Due to the failure of the validated F-gene rRT-PCR assay [24] and pigeon-specific fusion protein gene assay [39] to detect some of the genotype VI viruses studied here, the probe and the forward primer described by Kim et al., [39] were optimized as follows: F-4876 5′–[6-FAM] AAG CGY TTC TGT CTC YTT CCT CCT [BHQ_1]–3′ and (F + 4837) 5′- TGA TTC CAT CCG CAG GAT ACA AG -3′. Additionally, the reverse primer for the pigeon-specific F-gene assay (F-4837) was replaced with a new primer (F-4943) 5′- GCT GCT GTT ATC TGT GCC GA-3′. The optimized probe and newly designed primers were analyzed by OligoAnalyzer 3.1. (Integrated DNA Technologies, USA, https://eu.idtdna.com/calc/analyzer) tool and checked for self-annealing, hairpin loops and heterodimers. The sequences of the optimized primers and probes along with the tested fifteen NDV and selected representatives from other genotypes were aligned and compared to the M-gene and F-gene primers and probes and the pigeon-specific fusion probe to identify variable sites that determined the different outcomes of the F-gene rRT-PCR assay [24, 39] (see Additional file 2: Figs. S1, S2, and S3).

## Results

### Virus isolation and biological properties

Twenty-four NDV isolates from pigeons, chickens and quail, isolated in five different countries were studied here (Table 1). Most samples were obtained from pigeons (pet pigeons living in lofts, *n =* 12; zoo pigeons, *n =* 2; live bird markets, *n* *=* 2; and pigeons of unknown habitats, *n =* 6). Out of 31 oropharyngeal and cloacal Egyptian swabs from healthy birds inoculated in eggs, six contained live NDV and were fully characterized. The three Pakistani clinical samples were found to be positive for NDV by virus isolation and HI. All viruses obtained from repositories were successfully re-isolated after propagation and confirmed as NDV by HI. The ICPI values of selected viruses (pigeon/Egypt/Giza/11/2015, pigeon/Ukraine/Doneck/3/2007 and pigeon/Pakistan/Lahore/25A/2015) were 1.31, 1.48 and 1.46, respectively, and these indexes are typical for NDV with moderate virulence (mesogenic) in chickens [28].

**Table 1:** Background information data for Newcastle disease viruses isolated in Egypt, Ukraine, Pakistan, Bulgaria and South Korea analyzed in this study

Isolate name	Collection year	Country	Location	Host		Health status	Husbandry	Flock size	Age	Sequence coverage	Cleavage site motif (positions 113–117)	Genotype	GenBank acc.#
				Scientific name	Common name								
Giza/11	2015	Egypt	Giza	*Columba livia*	pigeon	apparently healthy	live bird market	NA	NA	complete genome	RQKR↓F	VI g	KY042129
Helwan/44	2015	Egypt	Helwan	Columba livia	pigeon	apparently healthy	live bird market	NA	NA	complete genome	RQKR↓F	VI g	KY042130
Qena/56	2015	Egypt	Qena	Columba livia	pigeon	apparently healthy	pet	200	2 years	complete genome	RQKR↓F	VI g	KY042131
El Fayom/73	2015	Egypt	El Fayom	Columba livia	pigeon	apparently healthy	pet	NA	NA	complete genome	RQKR↓F	VI g	KY042132
El Fayom /79	2015	Egypt	El Fayom	Columba livia	pigeon	apparently healthy	pet	NA	NA	complete genome	RQKR↓F	VI g	KY042133
El Fayom/84	2015	Egypt	El Fayom	Columba livia	pigeon	apparently healthy	pet	NA	NA	complete genome	RQKR↓F	VI g	KY042134
Ukraine/Kharkiv/2301	2013	Ukraine	Kharkiv	*Columba livia*	pigeon	dead/sick	pet	NA	2 years	complete genome	RQKR↓F	VI g	KY042127
Ukraine/Doneck/3	2007	Ukraine	Doneck	*Columba livia*	pigeon	dead/sick	pet	NA	2 years	complete genome	RQKR↓F	VI g	KY042128
Jhang/115^a^	2015	Pakistan	Jhang	*Columba livia*	pigeon	dead/sick	pet	110	2 year	full fusion	RKKR↓F	VI g	KY042137
Lahore/125^a^	2015	Pakistan	Lahore	*Columba livia*	pigeon	dead/sick	pet	200	1.5 years	full fusion	RKKR↓F	VI g	KY042136
Lahore/126^a^	2015	Pakistan	Lahore	*Columba livia*	pigeon	dead/sick	pet	200	1.5 years	full fusion	RKKR↓F	VI g	KY042138
Lahore/146^a^	2016	Pakistan	Lahore	*Columba livia*	pigeon	dead/sick	pet	55	1 year	full fusion	RKKR↓F	VI g	KY042139
Lahore/AW-1^a^	2014	Pakistan	Lahore	*Columba livia*	pigeon	dead	wildlife	39	2 years	full fusion	RQRR↓F	VI m	KU862297
Lahore/AW-2^a^	2015	Pakistan	Lahore	*Columba livia*	pigeon	dead/sick	pet	25	2 years	full fusion	RQRR↓F	VI m	KU862298
Lahore/AW-3^a^	2015	Pakistan	Lahore	*Columba livia*	pigeon	dead/sick	pet	200	3 months	full fusion	RQRR↓F	VI m	KU862299
Jallo-Lahore/221A^a^	2016	Pakistan	Lahore	*Columba livia*	pigeon	sick	zoo	20	2 years	full fusion	RQKR↓F	VI m	KY042140
Jallo-Lahore/221B^a^	2016	Pakistan	Lahore	*Columba livia*	pigeon	sick	zoo	20	2 years	full fusion	RQKR↓F	VI m	KY042141
Lahore/21A	2015	Pakistan	Lahore	*Columba livia*	pigeon	NA	NA	NA	NA	complete genome	RQKR↓F	VI m	KX236100
Lahore/22A	2015	Pakistan	Lahore	*Columba livia*	pigeon	dead/sick	pet	30	8 months	complete genome	RQKR↓F	VI m	KY042135
Lahore/25A	2015	Pakistan	Lahore	*Columba livia*	pigeon	NA	pet	20	1 year	complete genome	RQKR↓F	VI m	KX236101
Mokresh	1982	Bulgaria	Mokresh	*Gallus gallus*	chicken	NA	NA	NA	NA	complete genome	RQKR↓F	VI	KY042126
Dolnolinevo	1992	Bulgaria	Dolno Linevo	*Gallus gallus*	chicken	NA	NA	NA	NA	complete genome	RQKR↓F	VI c	KY042125
93-58GG	1993	South Korea	NA	*Gallus gallus*	chicken	NA	broiler farm	NA	14 days	full fusion	RRKR↓F	VI c	KY042142
88 M	1988	South Korea	NA	*Coturnix coturnix*	quail	NA	NA	NA	NA	full fusion	RRKR↓F	VI c	KY042143

^a^ these viruses were studied in Pakistan at the Quality Operations Laboratory (QOL) of the University of Veterinary & Animal Sciences (UVAS)

NA = not available

### Complete fusion protein gene and complete genome sequencing

The complete genome sequences of thirteen of the studied viruses were obtained at SEPRL, and the genome characteristics of these complete genomes are typical for NDV (See Additional file 1: Table S4). Additional eleven complete fusion gene coding sequences were also obtained and utilized for prediction of virulence and phylogenetic analysis. The fusion protein cleavage site of NDV is a major virulence determinant marker [40], and the deduced amino acid sequences of the fusion protein cleavage site revealed that all 24 isolates had multiple basic amino acids at positions 113–116, and a phenylalanine residue at position 117 (Table 1). The cleavage site motif of the Egyptian and Ukrainian viruses was ^113^RQKR↓F^117^ (*n =* 8). The Pakistani viruses (*n =* 12) had the following three motifs, ^113^RKKRF^117^, ^113^RQRR↓F^117^, and ^113^RQKR↓F^117^, sharing the last motif with the Bulgarian viruses (*n =* 2). The South Korean (*n* *=* 2) viruses had a ^13^RRKR↓F^117^ motif (Table 1). All of these motifs are characteristic of virulent NDV [3, 41].

### Distance and phylogenetic analyses

**Fig. 1:**
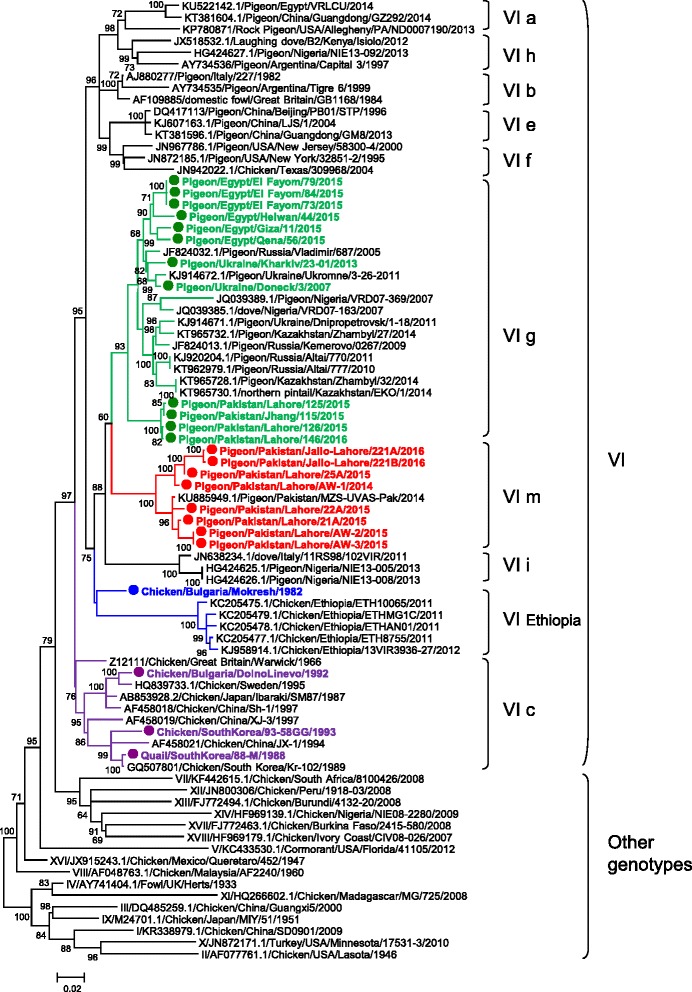
Phylogenetic analysis based on the complete nucleotide sequence of the fusion gene of viruses representing Newcastle disease viruses of class II. Only bootstrap values greater or equal to 60% are visualized. There were a total of 1650 positions in the final dataset. The strains sequenced in this study are highlighted in bold font and have a circle symbol in front the taxa name. Provisional designation of genotypes is indicated on the right

In order to determine the phylogenetic relationship between the studied viruses and other NDV isolated worldwide, the complete fusion gene coding sequences obtained in the current work, along with sequences of highly related viruses, were used to construct a phylogenetic tree (*n =* 82) (Fig. 1 and Additional file 1: Table S2). The full fusion coding region of all available class II NDV and of genotype VI (*n =* 1430 and *n =* 281, respectively, both including the 24 sequences from the current study) was also used to estimate evolutionary distances (Tables 2 and 3). A second phylogenetic analysis was performed using 83 complete genome-concatenated coding sequences of viruses pertaining to class II (see Additional file 1: Table S3 and Additional file 2: Fig. S4). Both, the full fusion gene and the complete genome phylogenetic analyses displayed similar topology confirming the phylogenetic classification of the viruses studied here into different sub-genotypes related to viruses previously isolated in Asia, Africa and Eastern Europe. The phylogenetic analyses (Fig. 1 and Additional file 2: Fig. S4) demonstrated that these 24 NDV studied here the clustered with viruses of genotype VI. The isolates from pigeons grouped with previously described viruses from columbine birds, while the chicken isolates grouped with other viruses from chickens. The topology of the full fusion phylogenetic tree (Fig. 1) indicated the viruses from Egypt (*n =* 6) (0%–3.1% genetic distance among themselves) and Ukraine (*n =* 2) (1.9% genetic distance between themselves) isolated from pigeons grouped together and clustered within sub-genotype VIg. These viruses were closely related to viruses isolated from pigeons in Russia, Ukraine, Kazakhstan, and Nigeria during 2005–2014 (Fig. 1) [42–44]. These results were consistent with the results of the complete genome analysis (see Fig. S4). Four Pakistani viruses isolated during 2015–2016 clustered together in a separate monophyletic branch within sub-genotype VIg (Fig. 1). The genetic distance of these Pakistani viruses (4.7%) to the rest of the VIg viruses shows higher nucleotide diversity within the sub-genotype.

**Table 2:** Еvolutionary distances^a^ between class II Newcastle disease virus of genotype VI estimated using the complete fusion gene coding sequences

Sub-genotype (number of analyzed sequences)	VI a	VI b	VI c	VI e	VI f	VI g	VI h	VI i	VI Ethiopia
VI a (*n* = 165)									
VI b (*n* = 10)	0.071								
VI c (*n* = 19)	0.091	0.069							
VI e (*n* = 16)	0.080	0.063	0.083						
VI f (*n* = 16)	0.075	0.058	0.082	0.059					
VI g (*n* = 25)	0.096	0.080	0.088	0.097	0.091				
VI h (*n* = 9)	0.071	0.071	0.098	0.084	0.081	0.103			
VI i (*n* = 4)	0.113	0.100	0.104	0.108	0.106	0.093	0.120		
VI Ethiopia (*n* = 8)	0.125	0.099	0.106	0.119	0.119	0.107	0.123	0.125	
VI m (*n* = 9)	0.111	0.091	0.099	0.112	0.101	0.085	0.112	0.107	0.120

^a^ The numbers of base substitutions per site from averaging over all sequence pairs between groups within genotype VI are shown. The analysis involved 281 nucleotide sequences. There were a total of 1647 positions in the final dataset

**Table 3:** Еvolutionary distances^a^ between class II Newcastle disease virus genotypes and sub-genotype VIm estimated using the complete fusion gene coding sequences

Genotype (number of analyzed sequences)	I	II	III	IV	V	VI	VII	VIII	IX	X	XI	XII	XIII	XIV	XV	XVI	XVII	XVIII
I (*n* = 139)																		
II (*n* = 171)	0.125																	
III (n = 10)	0.117	0.143																
IV (*n* = 5)	0.104	0.130	0.084															
V (*n* = 90)	0.191	0.206	0.178	0.148														
VI (*n* = 272)	0.185	0.204	0.177	0.136	0.159													
VII (*n* = 476)	0.180	0.213	0.168	0.139	0.159	0.132												
VIII (*n* = 5)	0.144	0.165	0.133	0.100	0.129	0.119	0.122											
IX (*n* = 35)	0.107	0.127	0.093	0.079	0.169	0.170	0.164	0.125										
X (*n* = 11)	0.115	0.114	0.141	0.129	0.207	0.201	0.199	0.164	0.125									
XI (*n* = 14)	0.201	0.224	0.191	0.130	0.229	0.234	0.239	0.197	0.172	0.224								
XII (*n* = 9)	0.192	0.224	0.179	0.152	0.169	0.129	0.118	0.126	0.177	0.205	0.248							
XIII (*n* = 44)	0.186	0.216	0.178	0.146	0.165	0.140	0.117	0.126	0.167	0.204	0.233	0.112						
XIV (*n* = 56)	0.221	0.260	0.221	0.184	0.191	0.167	0.143	0.155	0.216	0.235	0.283	0.134	0.139					
XV (*n* = 6)	0.145	0.132	0.135	0.109	0.163	0.145	0.112	0.127	0.108	0.159	0.210	0.149	0.144	0.178				
XVI (*n* = 4)	0.173	0.199	0.169	0.129	0.168	0.165	0.169	0.129	0.161	0.189	0.233	0.170	0.166	0.199	0.167			
XVII (*n* = 56)	0.182	0.222	0.187	0.152	0.171	0.151	0.132	0.138	0.174	0.212	0.235	0.124	0.120	0.132	0.155	0.184		
XVIII (*n* = 18)	0.192	0.217	0.185	0.153	0.172	0.139	0.125	0.136	0.176	0.209	0.236	0.119	0.115	0.134	0.148	0.179	0.109	
VI m (*n* = 9)	0.193	0.218	0.185	0.158	0.176	0.112	0.143	0.134	0.182	0.222	0.242	0.148	0.155	0.182	0.155	0.184	0.173	0.157

^a^ The numbers of base substitutions per site from averaging over all sequence pairs between genotypes are shown. The analysis involved 1430 nucleotide sequences. There were a total of 1596 positions in the final dataset

The remaining eight Pakistani viruses (0%–3.6% genetic distance among themselves, see Additional file 1: Table S5) isolated from pigeons during 2014–2016 did not cluster within any of the previously known sub-genotypes within genotype VI. These Pakistani isolates grouped with a Pakistani virus isolated from pigeon (KU885949/pigeon/Pakistan/MZS-UVAS/2014) creating a separate branch in the phylogenetic tree (Fig. 1) and were 8.5% to 12% distant from the rest of the sub-genotypes in genotype VI (Table 2). This new group of viruses fulfills all classification criteria set by Diel et al. [12] and was named as a novel, hitherto undescribed, sub-genotype of class II genotype VI, namely sub-genotype VIm. The viruses from the newly designated sub-genotype VIm were also more than 10% (11.2–24.2%) distant from all known NDV class II genotypes (Table 3).

The topology of the phylogenetic tree based on the full fusion gene sequences revealed that the Bulgarian virus (Chicken/Bulgaria/Mokresh/1982) grouped together with isolates from Ethiopia (during 2011–2012) [13] (Fig.1). However, the Bulgarian isolate was quite divergent from other members in this sub-genotype showing high genetic distance (10–11%). When this Bulgarian isolate was phylogenetically analyzed based on complete genome, it shared common ancestor with sub-genotypes VIg and VIm described above (see Additional file 2: Fig. S4). The other Bulgarian virus (chicken/Bulgaria/Dolno Linevo/1992), as well as both South Korean viruses obtained from a chicken and a quail, grouped within sub-genotype VIc with other chicken isolates from Sweden, Japan and China indicating that they belong to the older genotype VI sub-genotype (Fig. 1).

### Rapid diagnostic and development of new primers and probe

While all tested viruses were positive with the M-gene rRT-PCR assay [24], the F-gene rRT-PCR assay used to identify virulent NDV [24] failed to detect eight viruses from Egypt (*n =* 6) and Ukraine (*n =* 2) (Table 4). The pigeon-specific F-gene assay [39] failed to detect all fifteen NDV (the viruses sequenced in Pakistan were not tested, designated in Table 1). The genomic sequences of the viruses that failed detection were used to design new primers and probe based on a previous assay [39]. The new pigeon-specific F-gene test successfully detected all the genotype VI NDV (*n =* 15) that were not detected by the previously available protocol (Table 4). Upon further analysis, the new pigeon-specific fusion gene rRT-PCR assay was evaluated utilizing total RNA from allantoic fluids infected with virulent viruses of different genotypes and also side by side comparison of equal amount of total RNAs were performed with the M-gene and F-gene assays [24]. The results revealed that while the new fusion test recognized most effectively viruses of sub-genotype VIg, although with higher Ct values, it also recognized virulent viruses of seven different genotypes (II, Vb, VIa, VIIi, XII, XIVb, and XVIIa) (Table 4). While three Pakistani viruses (pigeon/Pakistan/Lahore/21A/2015, pigeon/Pakistan/Lahore/22A/2015 and pigeon/Pakistan/Lahore/25A/2015) were detected by the regular F-gene assay, using the new set of primers and probe resulted in four to nine lower Ct values (Table 4).

**Table 4:** Results of testing selected^a^ Newcastle disease viruses analyzed in this study by real-time RT-PCR using different sets of primers and probes

Isolate name	sub/genotype	Cycle threshold (Ct) values^b^		
		Matrix gene test^c^	Fusion-specific gene test^d^	New pigeon-specific fusion gene primers and probe (annealing temperature 56 °C)
pigeon/Egypt/Giza/11/2015	VI g	16.12	0	13.55
pigeon/Egypt/Helwan/44/2015	VI g	12.07	0	20.27
pigeon/Egypt/Qena/56/2015	VI g	12.27	0	15.92
pigeon/Egypt/El Fayom/73/2015	VI g	12.11	0	19.97
pigeon/Egypt/El Fayom /79/2015	VI g	14.72	0	15.57
pigeon/Egypt/El Fayom/84/2015	VI g	12.42	0	14.34
pigeon/Ukraine/Kharkiv/2301/2013	VI g	15.53	0	18.4
pigeon/Ukraine/Doneck/3/2007	VI g	12.11	0	21.62
pigeon/Pakistan/ Lahore/21A/2015	VI m	12.03	23.35	19.66
pigeon/Pakistan/Lahore/22A/2015	VI m	12.99	26.92	22.45
pigeon/Pakistan/Lahore/25A/2015	VI m	16.38	26.49	17.81
chicken/Bulgaria/Mokresh/1982	VI	13.72	24.25	21.83
chicken/Bulgaria/Dolnolinevo/1992	VI c	12.94	18.3	15.54
chicken/South Korea/9358GG/1993	VI c	12.31	26.61	24.84
quail/South Korea /88 M/1988	VI c	12.08	16.51	24.52
hawk/Mexico 663-ZM03/2008 (KC808489.1)	II	18.52	0	38.85
chicken/Belize/4224–3/08 (JN872163.1)	V b	16.23	18.95	28.02
parrot/Israel/2012/841 (KF792020.1)	VI a	16.63	26.41	32.42
chicken/KY-Israel/2013/50 (KF792019.1)	VII i	13.35	26.29	33.29
poultry/Peru/1918–03/2008 (JN800306.1)	XII	13.16	20.31	35.68
NG-707/GM.GMM.17-18 T (KC568207.1)	XIV b	12.29	17.07	32.05
chicken/DominicanRepublic499–31/2008 (JX119193.1)	XVI	17.44	17.92	0
NG-694/YB.GSH1.9-10C (KC568215.1)	XVII a	13.67	17.18	29.33

^a^ some Pakistani viruses were characterized only in Pakistan and were not submitted to SEPRL for further studies (designated in Table 1)

^b^ the pigeon-specific F-gene assay [39] failed to detect all fifteen NDV (the viruses sequenced in Pakistan were not tested)

^c,d^ primers and probes previously described by Wise et al. [24]

## Discussion

Here, we present the relationship between NDV of genotype VI isolated from Egypt, Ukraine, Pakistan, Bulgaria and South Korea based on their full fusion and complete genome characterization. Our data confirm the concurrent evolution and mobility of viruses of two sub-genotypes of genotype VI NDV across 3 continents. Viruses of genotype VI were first isolated from pigeons in the Middle East in 1960s and spread rapidly throughout Northern Africa to Europe and the rest of the world [45–47]. In Egypt, NDV was first identified in 1947 [48] on the basis of virus isolation into ECE and serologically by HI tests. Clinical signs in pigeons consistent with ND have been seen in Egypt since early 1981, and infection with NDV was serologically confirmed in diseased pigeons in the delta area of the Nile in 1984 [49, 50]. In Bulgaria, ND was first detected in 1943 [51], however the viruses of genotype VI were first found in Bulgaria in the mid-1970s [45]. To the best of our knowledge, the first identification of genotype VI NDV in Ukraine, Pakistan and South Korea has not been previously documented. The phylogenetic relationship among Eastern European, African and Asian viruses suggest the circulation of related viruses in pigeons across three continents.

Pigeons are not migratory and the circulation of these closely related viruses isolated from pigeons in six distant countries (Fig. 1) within eight years (2007–2015) is epidemiologically important. In many countries, pigeons (*Columba livia*) live freely as synantropic birds, or are bred for a variety of different purposes, such as a source of meat, pet companion birds, or for laboratory experiments in biology and cognitive science. Viruses of genotype VI have been reported previously to circulate in apparently healthy pigeons [52–54], and in the current work it was confirmed that at least sub-genotype VIg is seemingly maintained in healthy pigeons kept in captivity in Egypt (all samples from Egypt were collected from healthy pigeons). However, the mechanism of spread of these viruses at long distances remains unknown. A possible explanation for the spread of genotype VI NDV is the contact between columbid birds during competition flights, exhibitions, or due to the intensive international trade of such birds [55]. Other possibility is the international trade of live birds from other species or avian products between countries, either by legal or illegal routes of importation and exportation. In addition, NDV of genotype VI have also been isolated from birds from non-columbidae species kept in captivity and from wild birds, including partridges, pheasants, swans, falcons, blackbirds, cockatoos, budgerigars, raptors, partridges, crested ibises, waterfowl, starlings, pintails, gannets, and buzzards [44, 56–59]. However, while other NDV (e.g. genotype VII) are known to infect wild birds and this possibility can’t be excluded, no evidence exists that wild birds, especially migratory, play a role in the spread of genotype VI viruses. The presence of genotype VI viruses in fecal and oral swabs suggests that viral replication, which could result in virus transmission and possible outbreaks in poultry, as seen previously, is occurring in pigeons. Although sporadic, ND outbreaks in poultry caused by viruses of genotype VI have been reported [60–63], and the potential of the virus to cause clinical disease in poultry must not be under estimated.

The phylogenetic analyses revealed the complexity of NDV genotype VI and the challenges in the classification of its sub-genotypes. As shown in Table 2, some sub-genotypes have more than 10% distance compared to the rest of the sub-genotypes within genotype VI. In addition, the newly designated sub-genotype VIm is more than 10% distant from the rest of the genotypes in NDV class II (Table 3). These viruses topologically fall into a lower-order group (see Fig. 1 and Additional file 2: Fig. S4) compared to the existing genotype VI. Some of these groups of viruses meet, or will eventually meet the classification criteria for consideration as new genotypes. However, as naming new genotypes that originated from existing sub-genotypes may create confusion in the field of NDV classification, here, the most diverse group of viruses in genotype VI is named as sub-genotype VIm. In the future it may be appropriate to utilize rules similar to those put forth the WHO/OIE/FAO H5N1 Evolution Working Group for the nomenclature of highly pathogenic H5N1 avian influenza viruses [64] as already proposed by Susta et al. [65]. According to those rules, when a group is split into subgroups of higher order, the newly named groups remain part of the existing original lower-order group (e.g., VIa.1 or VIi.1 and so on). We believe that the criteria for naming new genotypes need to be updated; however, this has to be done based on international consensus rather than by individual scientific teams [65].

The failure of the validated F-gene rRT-PCT assay [24] designed to specifically detect viruses from the outbreak that occurred in southern California in 2002–2003 to detect the sub-genotype VIg Egyptian and the Ukrainian isolates (Table 4) was attributed to the mismatches in the probe and/or the primers. Here, several new variable sites that resulted in nucleotide mismatches were identified, suggesting continuous variation at the site used for the design of the test (see Additional file 2: Figsure S1, S2, and S3). The pigeon-specific probe, designed to detect dove/Italy/2736/2000 and US pigeon viruses [39] also had significant mismatches to the viruses tested here. The increasing number of mismatches of different probes and primers indicates that genotype VI is composed of a highly diverse group of viruses that is not covered by a single test. The newly developed pigeon test successfully identified the genotype VI isolates tested here (including the sub-genotype VIg); however, the new fusion test is not genotype specific, and positive detection of viruses of other genotypes is also possible (although with decreased sensitivity). Albeit also dependent on specific primers and not routinely performed in all laboratories, sequencing of the fusion gene will provide data allowing definitive diagnostics and characterization.

## Conclusion

In summary, genotype VI NDV continue to evolve in Africa, Asia and Europe suggesting the need for a constant surveillance and characterization of these viruses. The described epidemiological connections among viruses isolated from non-migratory birds on three continents creates uncertainty regarding the mechanism of spread of these viruses. Further studies are needed to elucidate this issue and understand the possible role of human activity in the dispersal of these viruses. Complete genomic characterization identified previously unrecognized genetic diversity that contributes to diagnostic failure and will facilitate future evolutionary studies. These results highlight the importance of updating existent rapid diagnostic protocols to detect emerging viral variants and help manage the disease in affected regions. The obtained rRT-PCR results suggest that the developed pigeon-specific F-gene assay, run in conjunction with the USDA-validated M-gene rRT-PCR assay, should be effective in detecting viruses from sub-genotype VIg, and those from the newly classified sub-genotype VIm.

## Additional files

Additional file 1: Table S1. Nucleotide sequences of primers used in PCR amplification, and sequencing of the NDV isolates used in this study. **Table S2.** List of the NDV used for construction of full fusion phylogenetic tree presented in Fig. 1. Highlighted in bold font are the viruses studied in the current work. **Table S3.** List of the NDV used for construction of complete genome phylogenetic tree presented in Additional file 2: Fig. S4. Highlighted in bold font are the viruses studied in the current work. **Table S4.** Characteristics of the thirteen complete genomes of Newcastle disease viruses of genotype VI sequenced in this study. **Table S5.** Estimated pairwise evolutionary distances among viruses of the new sub-genotype VIm. (DOCX 60 kb)

Additional file 2: Figure S1 A, B and C. Mismatches between the tested viruses and: **A)** previous fusion probe designed by Wise et al. [24]; **B)** pigeon-specific fusion probe designed by Kim et al. [66]; and **C)** the optimized pigeon specific probe in this study, respectively. Sequences are in order of 5′ to 3′. **Figure S2. A and B** Mismatches between the tested viruses and: **A)** previous fusion forward primer designed by Wise et al. [24]; and **B)** the optimized fusion forward primer in this study, respectively. Sequences are in order of 5′ to 3′. **Figure S3. A and B** Mismatches between the tested viruses and: **A)** previous fusion reverse primers designed by Wise et al. [24]; **B)** and the new fusion reverse in this study, respectively. Sequences are in order of 5′ to 3′. **Figure S4.** Phylogenetic analysis based on the complete genome concatenated coding sequence of viruses representing NDV class II. Only bootstrap values greater or equal to 60% are visualized. There were a total of 13,697 positions in the final dataset. The strains sequenced in this study are highlighted in bold font and have a circle symbol in front the taxa name. Provisional designation of genotypes is indicated on the right. (DOCX 1990 kb)
