# Supplementary material for: Phylogenetic assessment reveals continuous evolution and circulation of pigeon-derived virulent avian avulaviruses 1 in Eastern Europe, Asia, and Africa
Source: BMC Vet Res. 2017 Sep 26;13:291. doi: 10.1186/s12917-017-1211-4 (PMC5615457; doi:10.1186/s12917-017-1211-4)
Supplement: Supplementary file 1 — Nucleotide sequences of primers used in PCR amplification, and sequencing of the NDV isolates used in this study. Table S2. List of the NDV used for construction of full fusion phylogenetic tree presented in Fig. 1. Highlighted in bold font are the viruses studied in the current work. Table S3. List of the NDV used for construction of complete genome phylogenetic tree presented in Additional file 2: Fig. S4. Highlighted in bold font are the viruses studied in the current work. Table S4. Characteristics of the thirteen complete genomes of Newcastle disease viruses of genotype VI sequenced in this study. Table S5. Estimated pairwise evolutionary distances among viruses of the new sub-genotype VIm. (DOCX 60 kb) [file 12917_2017_1211_MOESM1_ESM.docx]

**Table S1**

Nucleotide sequences of primers used in PCR amplification, and sequencing of the NDV isolates used in this study.

| **Primers** | **5ʹ-3ʹ nucleotide sequence** | **Samples** | **Purpose** | | **Reference** |
| --- | --- | --- | --- | --- | --- |
| 1268_F | AAAYTAACTCCRGCAGCAAG | Pigeon /Egypt/Giza/11/2015 | Gaps closing | | This study |
| 1951_R | GACRGYTCCACTGGTCTC |  |  |  |  |
| 1312_F | ACGCGTATCTGAGGAGATTG | Pigeon/Ukraine/Doneck/3/2007 |  |  |  |
| 1896_R | CCATCTTCACTCTAGTCCACTG |  |  |  |  |
| 3069_F | CTGAATGGCTGATCACCATCA |  |  |  |  |
| 3661_R | GCTCTTCTTGCAAGTTATCAC |  |  |  |  |
| 1320 | CTGAAGAAATTGGCAGTG | Pigeon /Egypt/Helwan/44/2015  Pigeon/Egypt/Qena/56/2015  Pigeon/Egypt/El Fayom/73/2015  Pigeon/Egypt/El Fayom /79/2015  Pigeon/Egypt/El Fayom/84/2015 |  |  |  |
| 1897_R | GCCATCTTCACTCTAGTC |  |  |  |  |
| 19MER-2-5-6 | AGTACTGAGGCACATAACG | All | PCR | Termini sequencing | This study |
| 19MER-2-5-6-polyA(B) | AGTACTGAGGCACATAACGAAAAAAAAAAAAAAAAAAAAAB | All | RT primer after poly Utailing |  |  |
| 19MER-2-5-6-polyT(V) | AGTACTGAGGCACATAACGTTTTTTTTTTTTTTTTTTTTTV | All | RT primer after poly Atailing |  |  |
| 6F | TCATCCCRCTAAGGACATA | Pigeon/Ukraine/Doneck/3/2007  Pigeon/Ukraine/Kharkiv/2301/2013  Pigeon /Egypt/Giza/11/2015  Chicken/Bulgaria /Mokresh/1982 | PCRfor5’end |  | This study |
| 6F3 | CAGTACCTGCCCGAAGTATT | Pigeon/Egypt/El Fayom /79/2015  Pigeon /Egypt/Helwan/44/2015  Pigeon/Egypt/Qena/56/2015  Pigeon/Egypt/El Fayom/73/2015  Pigeon/Egypt/El Fayom/84/2015 |  |  | This study |
| 6R | CTATCTTCTGGATCATCRCT | Pigeon /Egypt/Giza/11/2015  Pigeon/Ukraine/Kharkiv/2301/2013  Chicken/Bulgaria /Mokresh/1982 | PCRfor3’end |  | This study |
| 6R3 | CTTCAACTCCAGCTGTAATG | Pigeon /Egypt/Helwan/44/2015  Pigeon/Egypt/Qena/56/2015  Pigeon/Egypt/El Fayom/73/2015  Pigeon/Egypt/El Fayom /79/2015  Pigeon/Egypt/El Fayom/84/2015 |  |  | This study |
| 6R4 | AAGAGACCCTGCTATCACCATG | Pigeon/Ukraine/Doneck/3/2007 |  |  |  |
| 4331F | GAGGTTACCTCYACYAAGCTRGAGA | The two South Korean samples: Chicken/Korea/93-58GG/1993 and Quail/Korea/88-M/1988 | For PCR and sanger sequencing | | (1) |
| 5090R | TCATTAACAAAYTGCTGCATCTTCCCWAC |  |  |  |  |
| MSF1 | GACCGCTGACCACGAGGTTA |  |  |  | (2) |
| NDVR2 | AGTCGGAGGATGTTGGCAGC |  |  |  |  |
| 4927F | TCTTGGGGTTGCAACAGCGGCAC |  |  |  | (3) |
| 5673R | GGCGTAGTGAGTGCACCTTCAG |  |  |  |  |
| 5491F | TGCCTCAGCACTTGTCCCGAAAG |  |  |  | (3) |
| 6341R | TCGATTGAAGGATGGCTCCTCTG |  |  |  |  |
| 4008F | ATATCGGGCTTATGTCCACTG | Pigeon/Pakistan/Jhang/115/2015  Pigeon/ Pakistan/Lahore/125/2015  Pigeon/Pakistan/Lahore/126/2015  Pigeon/Pakistan/Lahore/146/2016  Pigeon/Pakistan/Lahore/Aw-1/2014  Pigeon/Pakistan/Lahore/Aw-2/2015  Pigeon/Pakistan/Lahore/Aw-3/2015  Pigeon/Pakistan/Jallo-Lahore/221a/2016  Pigeon/Pakistan/Jallo-Lahore/221b/2016 | For PCR and sequencing by sanger | | (3) |
| 4994R | CTTAAGCCGGAGGATGTTGGC |  |  |  |  |
| NDV_8F | TTGACGGCAGGCCTCTTG |  |  |  | (4) |
| NDV_8R | GTGATAGAAGATCTTGACACCTC |  |  |  |  |
| NDV_9F | ATAATATGCGTGCCACCTA |  |  |  | (4) |
| NDV_9R | ATACACGGGTAGAACGGT |  |  |  |  |

**Table S2**

List of the NDV used for construction of full fusion phylogenetic tree presented in Fig. 1. Highlighted in bold font are the viruses studied in the current work.

| **Genotype** | **Accession #** | **Isolate description** | **Species** | **Year** | **Country** |
| --- | --- | --- | --- | --- | --- |
| I | KR338979.1 | chicken/China/SD0901/2009 | chicken | 2009 | China |
| II | AF077761.1 | Lasota | chicken | 1946 | USA |
| III | DQ485259 | chiken/China/Guangxi5/2000 | chicken | 2000 | china |
| IV | AY741404 | Herts | Fowl | 1933 | UK |
| V | KC433530.1 | Cormorant/Florida/41105/2012 | cormorant | 2012 | USA |
| VI a | KP780871.1 | ND0007190 | Rock Pigeon | 2013 | USA |
| VI a | KU522142 | pigeon/Egypt/VRLCU/2014 | pigeon | 2014 | Egypt |
| VI a | KT381604 | Pigeon/Guangdong/GZ292/2014 | pigeon | 2014 | China (Guangdong) |
| VI b | AF109885 | GB1168/84 | domestic fowl | 1984 | Great Britain |
| VI b | AY734535 | pigeon/Argentina/Tigre 6/99 | pigeon | 1999 | Argentina |
| VI b | AJ880277 | IT-227/82 | pigeon | 1982 | Italy |
| **VI c** | **KY042125** | **Chicken/Bulgaria/Dolnolinevo/1992** | **chicken** | **1992** | **Bulgaria** |
| **VI c** | **KY042142** | **Chicken/South Korea/93-58GG/1993** | **chicken** | **1993** | **South Korea** |
| **VI c** | **KY042143** | **Quail/South Korea /88M/1988** | **Quail** | **1988** | **South Korea** |
| VI c | AF458018 | Sh-1/97 | chicken | 1997 | China |
| VI c | HQ839733.1 | Chicken/Sweden/95 | chicken | 1995 | Sweden |
| VI c | AB853928.2 | APMV1/chicken/Japan/Ibaraki/SM87/1987 | chicken | 1987 | Japan |
| VI c | Z12111 | Warwick | chicken | 1966 | Great Britain |
| VI c | AF458019 | XJ-3/97 | chicken | 1997 | China |
| VI c | AF458021 | JX-1/94 | chicken | 1994 | China |
| VI c | GQ507801 | Kr-102/89 | chicken | 1989 | South Korea |
| VI e | DQ417113 | Pigeon/Beijing/PB01/STP/96 | pigeon | 1996 | China |
| VI e | KJ607163.1 | pi/CH/LJS/1/04 | pigeon | 2004 | China |
| VI e | KT381596.1 | Pigeon/Guangdong/GM8/2013 | pigeon | 2013 | China (Guangdong) |
| VI f | JN967786.1 | Pigeon/New Jersey/58300-4/2000 | pigeon | 2000 | USA |
| VI f | JN872185.1 | Pigeon/New York/32851-2/1995 | pigeon | 1984 | USA |
| VI f | JN942022.1 | PPMV Chicken/Texas/309968/2004 | chicken | 2004 | USA |
| **VI g** | **KY042129** | **Pigeon /Egypt/Giza/11/2015** | **pigeon** | **2015** | **Egypt** |
| **VI g** | **KY042130** | **Pigeon /Egypt/Helwan/44/2015** | **pigeon** | **2015** | **Egypt** |
| **VI g** | **KY042131** | **Pigeon/Egypt/Qena/56/2015** | **pigeon** | **2015** | **Egypt** |
| **VI g** | **KY042132** | **Pigeon/Egypt/El Fayom/73/2015** | **pigeon** | **2015** | **Egypt** |
| **VI g** | **KY042133** | **Pigeon/Egypt/El Fayom /79/2015** | **pigeon** | **2015** | **Egypt** |
| **VI g** | **KY042134** | **Pigeon/Egypt/El Fayom/84/2015** | **pigeon** | **2015** | **Egypt** |
| **VI g** | **KY042127** | **Pigeon/Ukraine/Kharkiv/2301/2013** | **pigeon** | **2013** | **Ukraine** |
| **VI g** | **KY042128** | **Pigeon/Ukraine/Doneck/3/2007** | **pigeon** | **2007** | **Ukraine** |
| **VI g** | **KY042137** | **Pigeon/Pakistan/Jhang/115/2015** | **pigeon** | **2015** | **Pakistan** |
| **VI g** | **KY042136** | **Pigeon/ Pakistan/Lahore/125/2015** | **pigeon** | **2015** | **Pakistan** |
| **VI g** | **KY042138** | **Pigeon/Pakistan/Lahore/126/2015** | **pigeon** | **2015** | **Pakistan** |
| **VI g** | **KY042139** | **Pigeon/Pakistan/Lahore/146/2016** | **pigeon** | **2016** | **Pakistan** |
| VI g | JF824013.1 | Pi/Rus/Kemerovo/0267/09 | pigeon | 2009 | Russia |
| VI g | JF824032.1 | Pi/Rus/Vladimir/687/05 | pigeon | 2005 | Russia |
| VI g | KJ920204.1 | Altai/pigeon/770/2011 | pigeon | 2011 | Russia |
| VI g | KT962979 | Pigeon/Russia/Altai/777/2010 | pigeon | 2010 | Russia |
| VI g | JQ039389.1 | pigeon/Nigeria/VRD07-369/2007 | pigeon | 2007 | Nigeria |
| VI g | JQ039385.1 | dove/Nigeria/VRD07-163/2007 | dove | 2007 | Nigeria |
| VI g | KJ914671.1 | Pigeon/Dnipropetrovsk/1-18-11 | pigeon | 2011 | Ukraine |
| VI g | KJ914672.1 | Pigeon/Ukromne/3-26-11 | pigeon | 2011 | Ukraine |
| VI g | KT965728.1 | pigeon /KZ/Zhambyl/32/2014 | pigeon | 2014 | Kazakhstan |
| VI g | KT965732 | pigeon/KZ/Zhambyl/27/2014 | pigeon | 2014 | Kazakhstan |
| VI g | KT965730 | anas acuta/KZ/EKO/1/2014 | northern pintail | 2014 | Kazakhstan |
| VI h | JX518532.1 | Laughing dove/B2/Kenya/Isiolo | laughing dove | 2012 | Kenya |
| VI h | HG424627.1 | pigeon/Nigeria/NIE13-092/2013 | pigeon | 2013 | Nigeria |
| VI h | AY734536 | pigeon/Argentina/Capital 3/97 | pigeon | 1997 | Argentina |
| VI i | JN638234.1 | DOVE/IT/11RS98/102VIR/2011 | dove | 2011 | Italy |
| VI i | HG424625.1 | pigeon/Nigeria/NIE13-005/2013 | pigeon | 2013 | Nigeria |
| VI i | HG424626.1 | pigeon/Nigeria/NIE13-008/2013 | pigeon | 2013 | Nigeria |
| **VI** | **KY042126** | **Chicken/Bulgaria/Mokresh/1982** | **chicken** | **1982** | **Bulgaria** |
| VI Eth | KC205475.1 | 2011/Ethiopia/ETH10065 | chicken | 2011 | Ethiopia |
| VI Eth | KC205477.1 | 2011/Ethiopia/ETH8755 | chicken | 2011 | Ethiopia |
| VI Eth | KC205478.1 | 2011/Ethiopia/ETHAN01 | chicken | 2011 | Ethiopia |
| VI Eth | KC205479.1 | 2011/Ethiopia/ETHMG1C | chicken | 2011 | Ethiopia |
| VI Eth | KJ958914.1 | APMV-1/Ethiopia/13VIR3936-27/2012 | chicken | 2012 | Ethiopia |
| **VI m** | **KU862297** | **Pigeon/Pakistan/Lahore/Aw1/2014** | **pigeon** | **2014** | **Pakistan** |
| **VI m** | **KU862298** | **Pigeon/Pakistan/Lahore/Aw2/2015** | **pigeon** | **2015** | **Pakistan** |
| **VI m** | **KU862299** | **Pigeon/Pakistan/Lahore/Aw3/2015** | **pigeon** | **2015** | **Pakistan** |
| **VI m** | **KY042140** | **Pigeon/Pakistan/Jallo-Lahore/221A/2016** | **pigeon** | **2016** | **Pakistan** |
| **VI m** | **KY042141** | **Pigeon/Pakistan/Jallo-Lahore/221B/2016** | **pigeon** | **2016** | **Pakistan** |
| **VI m** | **KX236100** | **Pigeon/Pakistan/ Lahore/21A/2015** | **pigeon** | **2015** | **Pakistan** |
| **VI m** | **KY042135** | **Pigeon/Pakistan/Lahore/22A/2015** | **pigeon** | **2015** | **Pakistan** |
| **VI m** | **KX236101** | **Pigeon/Pakistan/Lahore/25A/2015** | **pigeon** | **2015** | **Pakistan** |
| VI m | KU885949.1 | Pigeon/MZS-UVAS-Pak/2014 | pigeon | 2014 | Pakistan |
| VII d | KF442615.1 | chicken/South Africa/08100426/2008 | chicken | 2008 | South Africa |
| VIII | AF048763 | AF2240 | chicken | 1960 | Malaysia |
| IX | M24701.1 | MIY/51 | chicken | 1951 | Japan |
| X | JN872171.1 | Turkey/Minnesota/17531-3/2010 | turkey | 2010 | USA |
| XI | HQ266602 | MG/725/08 | chicken | 2008 | Madagascar |
| XII | JN800306 | poultry/Peru/1918-03/2008 | chicken | 2008 | Peru |
| XIII a | FJ772494 | chicken/4132-20/Burundi/2008 | chicken | 2008 | Burundi |
| XIV a | HF969139.1 | chicken/Nigeria/NIE08-2280/2009 | chicken | 2009 | Nigeria |
| XVI | JX915243.1 | chicken/Mexico/Queretaro/452/1947 | chicken | 1947 | Mexico |
| XVII a | FJ772463.1 | chicken/2415-580/Burkina Faso/2008 | chicken | 2008 | Burkina Faso |
| XVIII a | HF969179.1 | chicken/Ivory Coast/CIV08-026/2007 | chicken | 2007 | Ivory Coast |

**Table S3**

List of the NDV used for construction of complete genome phylogenetic tree presented in Fig. S4. Highlighted in bold font are the viruses studied in the current work.

| **Genotype** | **Accession #** | **Isolate description** | **Species** | **Year** | **Country** |
| --- | --- | --- | --- | --- | --- |
| I | AY935500 | I-2progenitor | chicken | 2005 | Australia |
| I | KT894018.1 | HuN123 | --- | 2014 | China |
| I | HM063422.1 | D3/Guang-dong/2007 | feralduck | 2007 | China |
| I | HM125898.1 | WDK/Jiangxi/7793/2004 | duck | 2004 | China |
| I | DQ097394 | vaccine strain/PHY-LMV42/66 | --- | 1966 | --- |
| I | GQ918280 | BHG/Sweeden/94 | black-headed gull | 1994 | Sweeden |
| I | AY562991 | Ulster | chicken | 1967 | Ireland |
| II | AY845400.2 | Lasota-AY | chicken | 1946 | USA |
| II | GU978777.1 | APMV-1/Chicken/U.S.(TX)GB/1948 | chicken | 1948 | USA |
| III | EF201805 | Mukteswar | avian | 1940 | --- |
| III | FJ430159 | JS/7/05 | chicken | 2005 | China |
| IV | AY741404 | Herts | Fowl | 1933 | UK |
| IV | EU293914 | Italien | --- | 1944 | Italy |
| V | GQ288381.2 | cormorant/US(CA)/D9704285/1997 | Cormorant | 1997 | USA |
| V | GQ288382.2 | cormorant/Canada/98CNN3-V1125/1998 | Cormorant | 1998 | Canada |
| V | KJ577136.1 | Chimalhuacan | chicken | 1973 | Mexico |
| V | KF767466.1 | Belize (Spanish Lookout)/4224-3/2008 | chicken | 2008 | Belize |
| V | AY562986 | anhinga/U.S.(Fl)/44083/93 | Anhinga | 1993 | USA |
| V | EF065682 | rAnhinga | Anhinga | --- | USA |
| VI a | AY562989.1 | dove/Italy/2736/00 | Dove | 2000 | Italy |
| VI a | KT163262.1 | Pi/SH/CH/0167/2013 | pigeon | 2013 | China |
| VI a | KC013038.1 | APMV1/Pigeon/PA/USA/0810/2008 | pigeon | 2008 | USA |
| VI a | KC013032.1 | APMV1/Pigeon/USA/0106/2001 | pigeon | 2001 | USA |
| VI a | FJ766526 | JS-07/22/Pi | pigeon | 2007 | China |
| VI a | JN986839.1 | PPMV-1/pigeon/IE/806/04 | pigeon | 2004 | Ireland |
| VI a | JX486550.1 | pi/CH/LGD/110208 | pigeon | 2011 | China |
| VI a | JX901110.1 | PPMV-1/Belgium/98-248/1998 | pigeon | 1998 | Belgium |
| VI a | JX901120.1 | PPMV-1/Belgium/05-03936-8/2005 | pigeon | 2005 | Belgium |
| VI a | KJ736742.1 | R75/98 | pigeon | 1998 | Germany |
| VI b | FJ410145 | PPMV-1/New York/1984 | pigeon | 1984 | USA |
| VI b | AJ880277 | IT-227/82 | pigeon | 1982 | Italy |
| VI b | FJ410147.1 | PPMV-1/Maryland/1984 | pigeon | 1984 | USA |
| VI c | HQ839733.1 | Chicken/Sweden/95 | chicken | 1995 | Sweden |
| VI c | AB853928.2 | APMV1/chicken/Japan/Ibaraki/SM87/1987 | chicken | 1987 | Japan |
| VI c | AB853926.2 | APMV1/chicken/Japan/Osaka/2440/1969 | chicken | 1969 | Japan |
| **VI c** | **KY042125** | **Chicken/Bulgaria/Dolnolinevo/1992** | **chicken** | **1992** | **Bulgaria** |
| VI c | FJ766529.1 | ZhJ-3/97 | chicken | 1997 | China |
| VI c | KC853020.1 | NDV/crested ibis/China/Shaanxi10/2010 | crested ibis | 2010 | China |
| VI e | FJ766528 | NDV/05/029 | pigeon | 2005 | China |
| VI e | GQ338311 | ND/05/028 | --- | 2005 | China |
| VI e | KJ607163.1 | pi/CH/LJS/1/04 | pigeon | 2004 | China |
| VI e | KJ607164.1 | pi/CH/LJS/1/03 | pigeon | 2003 | China |
| VI e | KJ808820.1 | Pigeon/China/SD2012 | pigeon | 2012 | China |
| VI f | KC013031.1 | APMV1/Pigeon/USA/0101/2001 | pigeon | 2001 | USA |
| VI f | KC013040.1 | APMV1/Pigeon/PA/USA/0712/2007 | pigeon | 2007 | USA |
| **VI g** | **KY042129** | **Pigeon /Egypt/Giza/11/2015** | **pigeon** | **2015** | **Egypt** |
| **VI g** | **KY042130** | **Pigeon /Egypt/Helwan/44/2015** | **pigeon** | **2015** | **Egypt** |
| **VI g** | **KY042131** | **Pigeon/Egypt/Qena/56/2015** | **pigeon** | **2015** | **Egypt** |
| **VI g** | **KY042132** | **Pigeon/Egypt/El Fayom/73/2015** | **pigeon** | **2015** | **Egypt** |
| **VI g** | **KY042133** | **Pigeon/Egypt/El Fayom /79/2015** | **pigeon** | **2015** | **Egypt** |
| **VI g** | **KY042134** | **Pigeon/Egypt/El Fayom/84/2015** | **pigeon** | **2015** | **Egypt** |
| **VI g** | **KY042127** | **Pigeon/Ukraine/Kharkiv/2301/2013** | **pigeon** | **2013** | **Ukraine** |
| **VI g** | **KY042128** | **Pigeon/Ukraine/Doneck/3/2007** | **pigeon** | **2007** | **Ukraine** |
| VI g | JF824013.1 | Pi/Rus/Kemerovo/0267/09 | pigeon | 2009 | Russia |
| VI g | JF824032.1 | Pi/Rus/Vladimir/687/05 | pigeon | 2005 | Russia |
| VI g | KT962979 | Pigeon/Russia/Altai/777/2010 | pigeon | 2010 | Russia |
| VI g | KJ920204.1 | Altai/pigeon/770/2011 | pigeon | 2011 | Russia |
| **VI** | **KY042126** | **Chicken/Bulgaria/Mokresh/1982** | **chicken** | **1982** | **Bulgaria** |
| **VI m** | **KX236100** | **Pigeon/Pakistan/ Lahore/21A/2015** | **pigeon** | **2015** | **Pakistan** |
| **VI m** | **KY042135** | **Pigeon/Pakistan/Lahore/22A/2015** | **pigeon** | **2015** | **Pakistan** |
| **VI m** | **KX236101** | **Pigeon/Pakistan/Lahore/25A/2015** | **pigeon** | **2015** | **Pakistan** |
| VI m | KU885949.1 | Pigeon/MZS-UVAS-Pak/2014 | pigeon | 2014 | Pakistan |
| VII | JQ015296.1 | Chicken/China/SD04/2011 | chicken | 2011 | China |
| VII | KC542893.1 | Chicken/China/Liaoning/02/2005 | chicken | 2005 | China |
| VII | AF431744.3 | ZJ1 | goose | 2000 | China |
| VII | KJ782375.1 | go/CH/GD-QY/1997 | goose | 1997 | China |
| VII | GQ338310 | ND/03/044 | pigeon | 2003 | China |
| VII | KP776462.1 | chicken/NDV/Pak/AW-14 | chicken | 2014 | Pakistan |
| VIII | FJ751918 | QH1 | chicken | 1979 | China |
| VIII | FJ751919 | QH4 | chicken | 1985 | China |
| IX | FJ436302 | F48E8 | chicken | 1946-1948 | China |
| IX | FJ436303 | ZJ/1/86/Ch | chicken | 1986 | China |
| X | FJ705469 | mallard/US(MN)/MN00-39/2000 | mallard | 2000 | USA |
| XI | HQ266602 | MG/725/08 | chicken | 2008 | Madagascar |
| XII | KC551967.1 | goose/Guangdong/2010 | goose | 2010 | China |
| XII | KR732614.1 | NDV/peacock/Peru/2011 | peacock | 2011 | Peru |
| XIII | AY865652 | Sterna/Astr/2755/2001 | Sterna albifrons | 2001 | Russia |
| XIII | KP089979.1 | Nagpur | chicken | 2012 | India |
| XVI | JX119193.1 | chicken/Dominican Republic(Juan-Lopez)/499-31/2008 | chicken | 2008 | Dominican Republic |
| XVII | JF966385.1 | chicken/2008/Mali/ML007/08 | chicken | 2008 | Mali |
| XVIII | JF966387.1 | chicken/2009/Mali/ML008 | chicken | 2009 | Mali |
| XVIII | JX546248.1 | chicken/Togo/AKO18/2009 | chicken | 2009 | Togo |

**Table S4**

Characteristics of the thirteen complete genomes of Newcastle disease viruses of genotype VI sequenced in this study^a^.

| Region | Gene start positions (length) | Length of 3ʹ UTR^b^ | Coding sequence positions (length)^c^ | Length of 5ʹ UTR | Gene end positions (length) | Length of intergenic regions | Length of the whole region | Amino acid length |
| --- | --- | --- | --- | --- | --- | --- | --- | --- |
| Leader | 1–55 (55) |  |  |  |  |  |  |  |
| NP | 56-65 (10) | 56 | 122-1591 (1470) | 206 | 1798-1808 (11) | 1 | 1753 | 489 |
| P | 1810-1819 (10) | 73 | 1893-3080 (1188) | 169 | 3250-3260 (11) | 1 | 1451 | 395 |
| M | 3262-3271 (10) | 24 | 3296-4390 (1095) | 102 | 4493-4502 (10) | 1 | 1241 | 364 |
| F | 4504-4513 (10 | 36 | 4550-6211 (1662) | 73 | 6285-6295 (11) | 31 | 1792 | 553 |
| HN | 6327-6336 (10) | 81 | 6418-8133 (1716) | 185 | 8319-8328 (10) | 47 | 2002 | 571 |
| L | 8376-8385 (10) | 1 | 8387-14001 (6615) | 67 | 15069-15078 (10) |  | 6703 | 2204 |
| Trailer | 15079-15192 (114) |  |  |  |  |  |  |  |
| Total length |  |  |  |  |  |  | 15192 |  |

All lengths are in nucleotides (except for the amino acids);

^a^ all thirteen genomes sequenced in this study had same genome length characteristics;

^b^UTR = untranslated regions;

^c^ including stop codon.

**Table S5**

Estimated pairwise evolutionary distances among viruses of the new sub-genotype VIm.

|  | Designated isolates | 1 | 2 | 3 | 4 | 5 | 6 | 7 | 8 |
| --- | --- | --- | --- | --- | --- | --- | --- | --- | --- |
| 1 | KU885949.1/pigeon/Pak/MZS-UVAS/2014 |  |  |  |  |  |  |  |  |
| 2 | Pigeon/Pak/Lahore/AW-1/2014 | 0.031 |  |  |  |  |  |  |  |
| 3 | Pigeon/Pak/Lahore/AW-2/2015 | 0.017 | 0.026 |  |  |  |  |  |  |
| 4 | Pigeon/Pak/Lahore/AW-3/2015 | 0.017 | 0.026 | 0.000 |  |  |  |  |  |
| 5 | Pigeon/Pak/Lahore/22A/2015 | 0.010 | 0.034 | 0.023 | 0.023 |  |  |  |  |
| 6 | Pigeon/Pak/Lahore/25A/2015 | 0.035 | 0.010 | 0.032 | 0.032 | 0.039 |  |  |  |
| 7 | Pigeon/Pak/Jallo-Lahore/221A/2016 | 0.052 | 0.025 | 0.046 | 0.046 | 0.055 | 0.014 |  |  |
| 8 | Pigeon/Pak/Lahore/21A/2015 | 0.007 | 0.035 | 0.012 | 0.012 | 0.014 | 0.039 | 0.056 |  |
| 9 | Pigeon/Pak/Jallo-Lahore/221B/2016 | 0.049 | 0.024 | 0.044 | 0.044 | 0.053 | 0.015 | 0.002 | 0.054 |

The numbers of base substitutions per site between sequences are shown. There were a total of 1662 positions in the final dataset.

1. **Kim LM, King DJ, Suarez DL, Wong CW, Afonso CL.** 2007. Characterization of class I Newcastle disease virus isolates from Hong Kong live bird markets and detection using real-time reverse transcription-PCR. J Clin Microbiol **45:**1310-1314. <http://dx.doi.org/10.1128/JCM.02594-06>

2. **Aldous EW, Mynn JK, Banks J, Alexander DJ.** 2003. A molecular epidemiological study of avian paramyxovirus type 1 (Newcastle disease virus) isolates by phylogenetic analysis of a partial nucleotide sequence of the fusion protein gene. Avian Pathol **32:**239-256. <http://dx.doi.org/10.1080/030794503100009783>

3. **Miller PJ, Dimitrov KM, Williams-Coplin D, Peterson MP, Pantin-Jackwood MJ, Swayne DE, Suarez DL, Afonso CL.** 2015. International biological engagement programs facilitate Newcastle disease epidemiological studies. Front Public Health **3:**235. <http://dx.doi.org/10.3389/fpubh.2015.00235>

4. **Munir M, Abbas M, Khan MT, Zohari S, Berg M.** 2012. Genomic and biological characterization of a velogenic Newcastle disease virus isolated from a healthy backyard poultry flock in 2010. Virol J **9:**46. 10.1186/1743-422X-9-46

**REFERENCES**
