# Supplementary material for: Phylogenetic assessment reveals continuous evolution and circulation of pigeon-derived virulent avian avulaviruses 1 in Eastern Europe, Asia, and Africa
Source: BMC Vet Res. 2017 Sep 26;13:291. doi: 10.1186/s12917-017-1211-4 (PMC5615457; doi:10.1186/s12917-017-1211-4)
Supplement: Supplementary file 2 — Mismatches between the tested viruses and: A) previous fusion probe designed by Wise et al. [24]; B) pigeon-specific fusion probe designed by Kim et al. [66]; and C) the optimized pigeon specific probe in this study, respectively. Sequences are in order of 5′ to 3′. Figure S2. A and B Mismatches between the tested viruses and: A) previous fusion forward primer designed by Wise et al. [24]; and B) the optimized fusion forward primer in this study, respectively. Sequences are in order of 5′ to 3′. Figure S3. A and B Mismatches between the tested viruses and: A) previous fusion reverse primers designed by Wise et al. [24]; B) and the new fusion reverse in this study, respectively. Sequences are in order of 5′ to 3′. Figure S4. Phylogenetic analysis based on the complete genome concatenated coding sequence of viruses representing NDV class II. Only bootstrap values greater or equal to 60% are visualized. There were a total of 13,697 positions in the final dataset. The strains sequenced in this study are highlighted in bold font and have a circle symbol in front the taxa name. Provisional designation of genotypes is indicated on the right. (DOCX 1990 kb) [file 12917_2017_1211_MOESM2_ESM.docx]

**
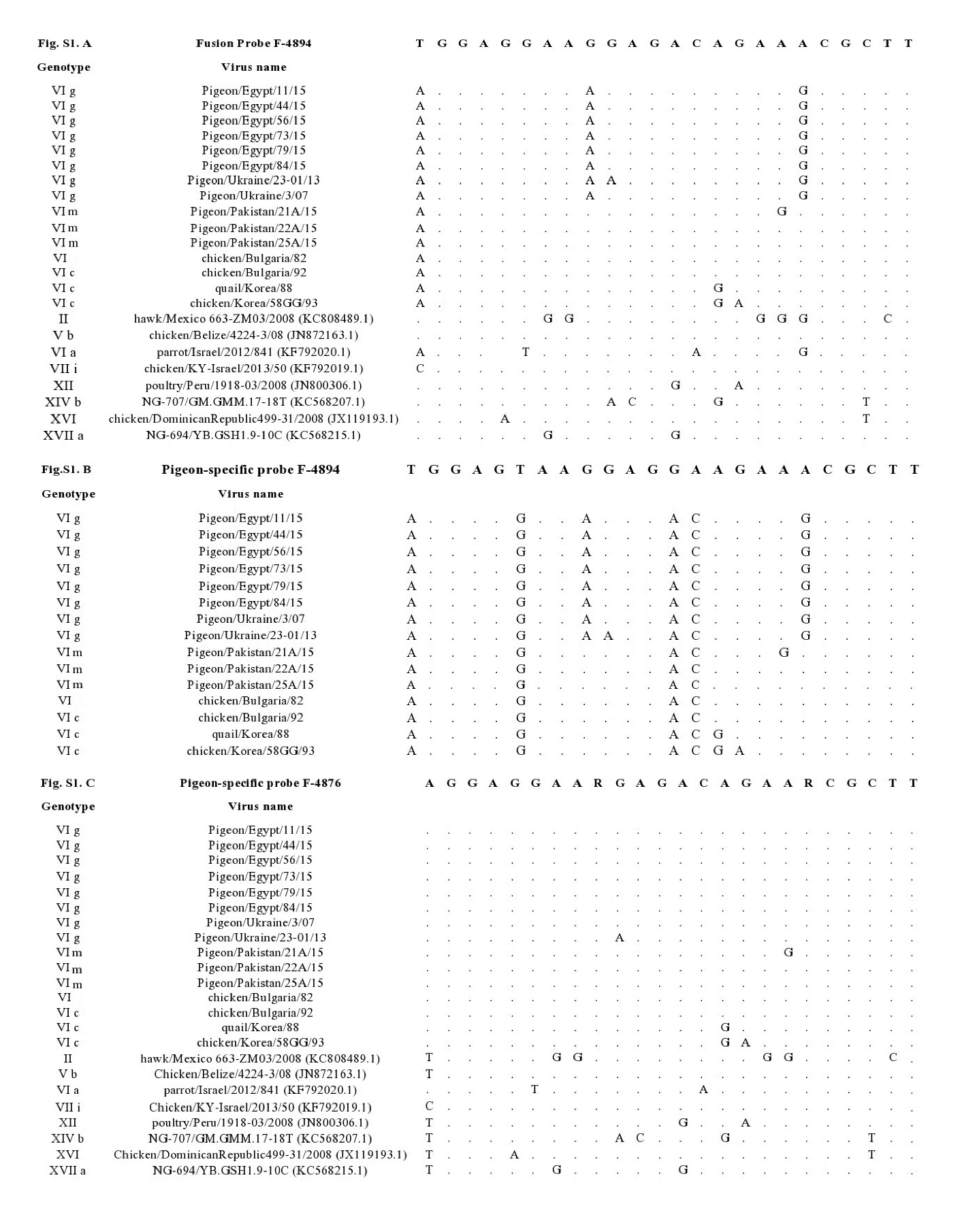
Figure S1 A, B and C**

Mismatches between the tested viruses and: **A)** previous fusion probe designed by Wise et al. (1); **B)** pigeon-specific fusion probe designed by Kim et al. (2); and **C)** the optimized pigeon specific probe in this study, respectively. Sequences are in order of 5ʹ to 3ʹ.


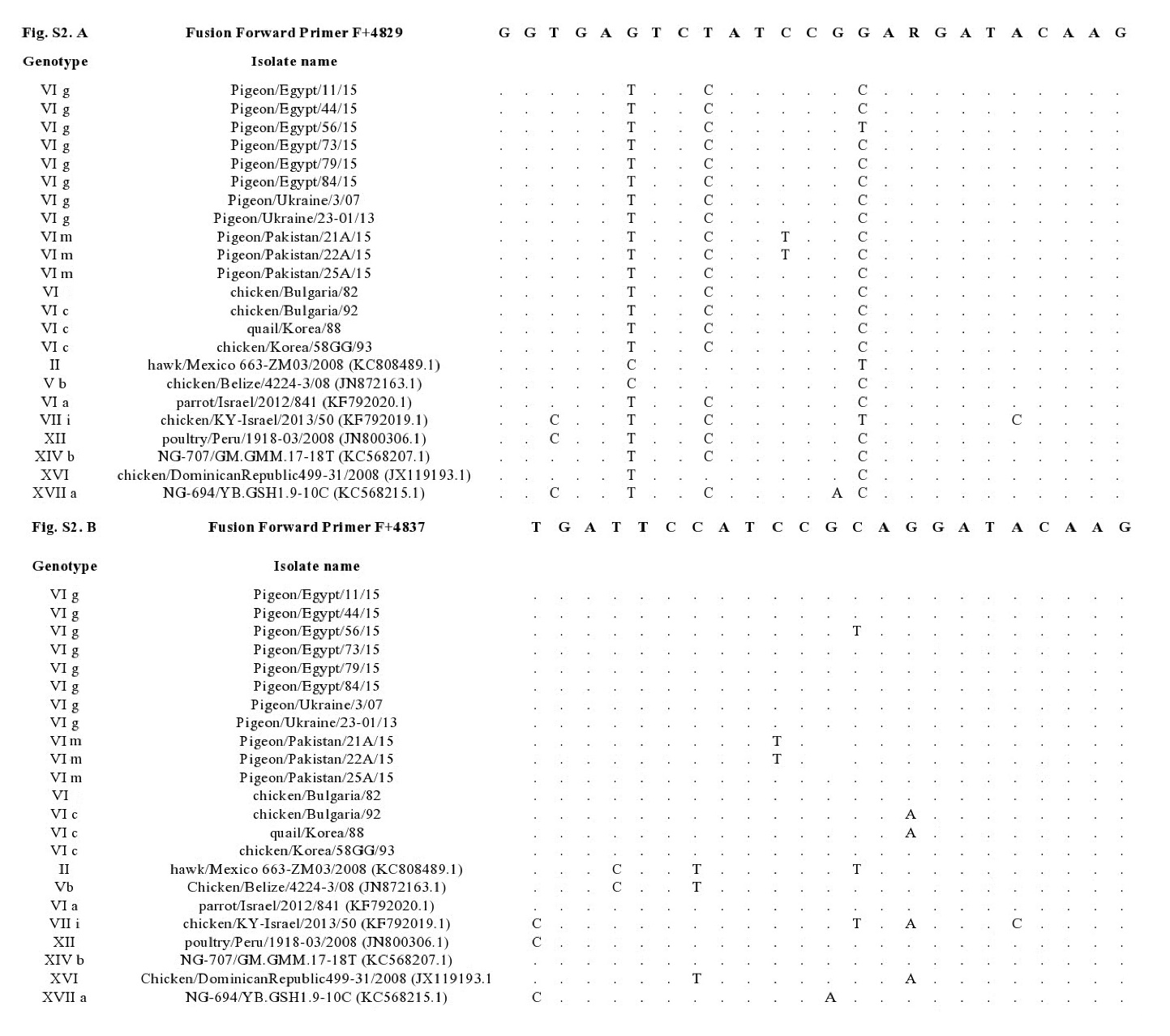


**Figure S2 A and B**

Mismatches between the tested viruses and: **A)** previous fusion forward primer designed by Wise et al. (1); and **B)** the optimized fusion forward primer in this study, respectively. Sequences are in order of 5ʹ to 3ʹ.


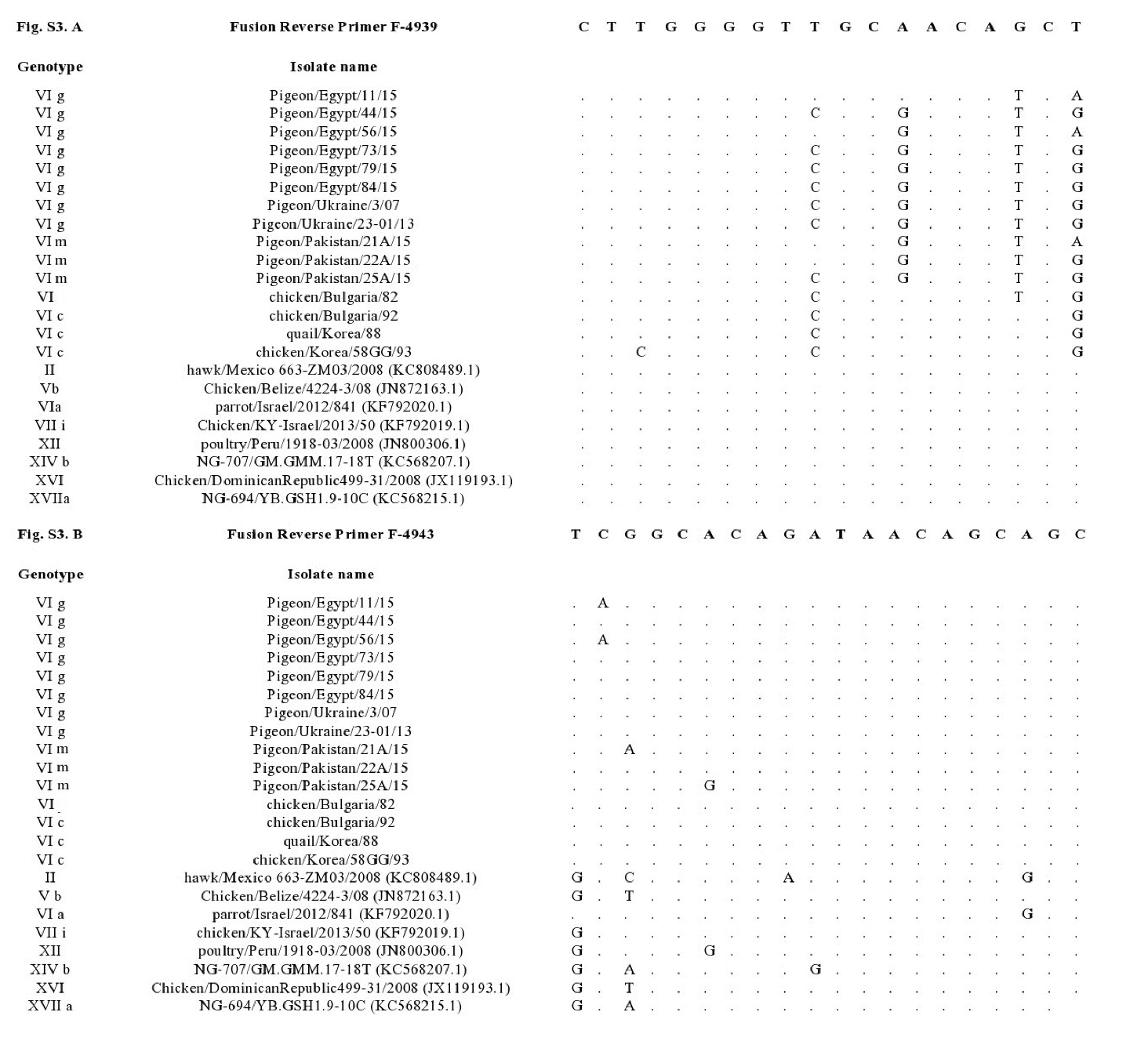


**Figure S3 A and B**

Mismatches between the tested viruses and: **A)** previous fusion reverse primers designed by Wise et al. (1); **B)** and the new fusion reverse in this study, respectively. Sequences are in order of 5ʹ to 3ʹ.


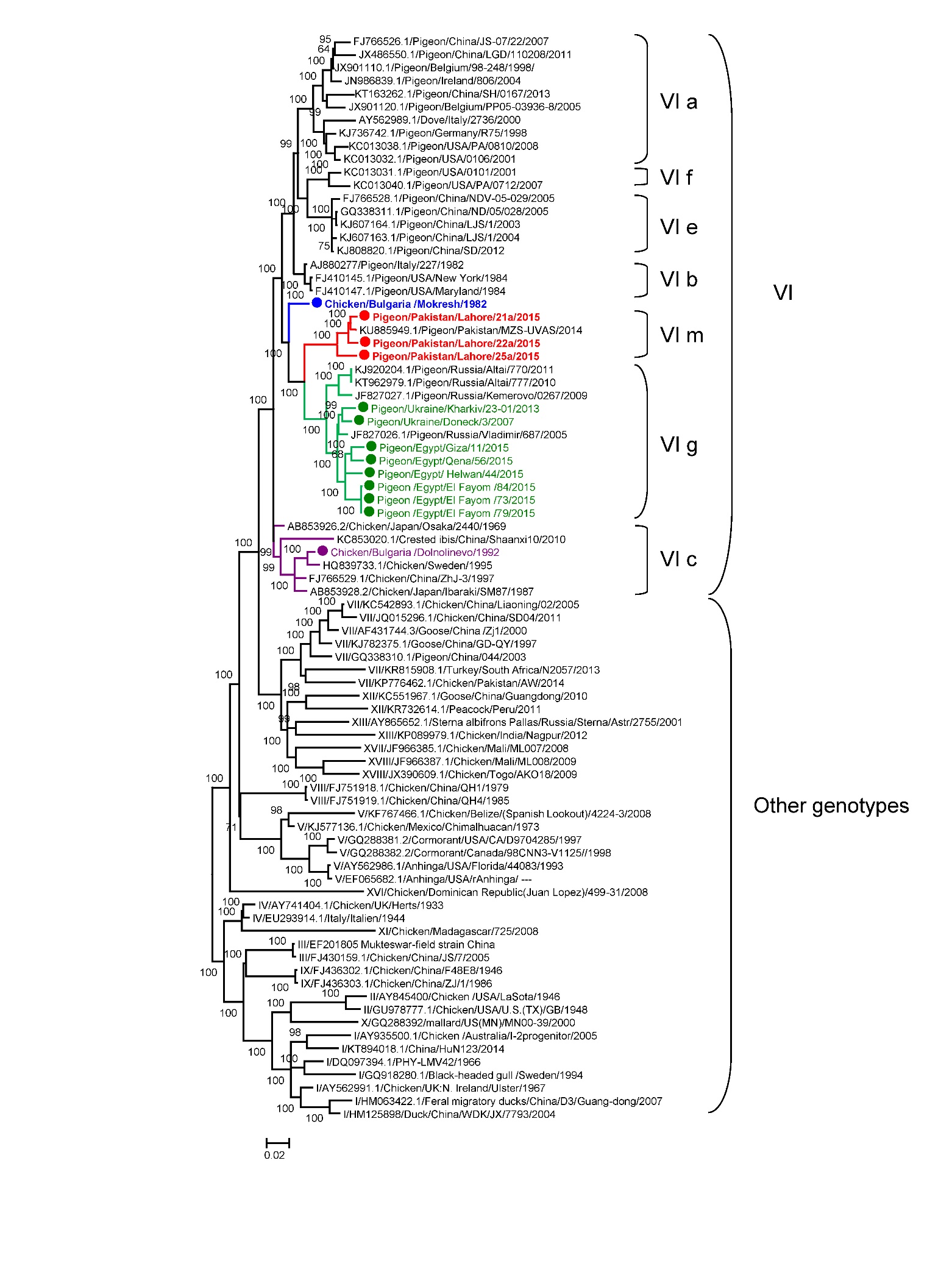


**Figure S4**

Phylogenetic analysis based on the complete genome concatenated coding sequence of viruses representing NDV class II. Only bootstrap values greater or equal to 60% are visualized. There were a total of 13697 positions in the final dataset. The strains sequenced in this study are highlighted in bold font and have a circle symbol in front the taxa name. Provisional designation of genotypes is indicated on the right.

1. **Wise MG, Suarez DL, Seal BS, Pedersen JC, Senne DA, King DJ, Kapczynski DR, Spackman E.** 2004. Development of a real-time reverse-transcription PCR for detection of newcastle disease virus RNA in clinical samples. J Clin Microbiol **42:**329-338. <http://dx.doi.org/10.1128/JCM.42.1.329-338.2004>

2. **Kim LM, King DJ, Suarez DL, Wong CW, Afonso CL.** 2007. Characterization of class I Newcastle disease virus isolates from Hong Kong live bird markets and detection using real-time reverse transcription-PCR. J Clin Microbiol **45:**1310-1314. <http://dx.doi.org/10.1128/JCM.02594-06>

REFERENCES
